# Role of Immunoglobulins in Muscular Dystrophies and Inflammatory Myopathies

**DOI:** 10.3389/fimmu.2021.666879

**Published:** 2021-07-14

**Authors:** Andrea Farini, Chiara Villa, Luana Tripodi, Mariella Legato, Yvan Torrente

**Affiliations:** Stem Cell Laboratory, Department of Pathophysiology and Transplantation, University of Milan, Dino Ferrari Center, Fondazione IRCCS Cà Granda Ospedale Maggiore Policlinico, Milan, Italy

**Keywords:** muscular dystrophies, immunoglobulins, autoimmunity, inflammatory myopathies, muscle inflammation, autoantibodies

## Abstract

Muscular dystrophies and inflammatory myopathies are heterogeneous muscular disorders characterized by progressive muscle weakness and mass loss. Despite the high variability of etiology, inflammation and involvement of both innate and adaptive immune response are shared features. The best understood immune mechanisms involved in these pathologies include complement cascade activation, auto-antibodies directed against muscular proteins or *de-novo* expressed antigens in myofibers, MHC-I overexpression in myofibers, and lymphocytes-mediated cytotoxicity. Intravenous immunoglobulins (IVIGs) administration could represent a suitable immunomodulator with this respect. Here we focus on mechanisms of action of immunoglobulins in muscular dystrophies and inflammatory myopathies highlighting results of IVIGs from pre-clinical and case reports evidences.

## Introduction

Growing evidences support the role of the immune system in different pathological conditions of the skeletal muscle. Immune cell infiltrate following muscle injury contributes to the pathology of various muscular dystrophies (MDs), whereas autoimmune responses specific for defined or yet undefined muscle antigens are suggested as the cause of some idiopathic inflammatory myopathies (IIMs). The initial immune response to muscle damage consists of innate immunity in which phagocytic, cytolytic, and secretory inflammatory cells (mainly macrophages and neutrophils) are rapidly mobilized and activated to identify, kill, and remove invading infectious organisms during infectious events or remove muscle fiber debris and promote muscle repair following disruption of muscle homeostasis. Innate immunity predates the adaptive immune system through the activation of professional antigen-presenting cells (APCs) that process and present muscle antigens to T-effector cells (mainly T-CD4+ and T-CD8+ cells) toward major histocompatibility complex (MHC) leading to intensive secretion of pro-inflammatory cytokines and muscle fiber necrosis (1). The MDs constitute a group of genetically muscle diseases characterized by progressive muscle weakness and degeneration. The most frequently occurring MDs involve damage to the muscle fiber membrane, which can lead to the release of Danger Associated Molecular Patterns (DAMPs) in the extracellular environment which interact with toll-like receptors (TLR) of APCs such as DCs and macrophages, triggering an innate immune response, with recruitment of inflammasomes and activation of the NF-kB signaling pathway. Among DAMPs released from damaged fiber there are Histidyl-tRNA synthetase (HRS) and High Mobility Group 1 Binding protein (HMGB1). The HMGB1 protein binds to TLR4 expressed on DCs and macrophages (2) becoming competent APCs that can activate T lymphocytes recognizing the antigen on their surface complexed to MHC molecules (3–5). The HMGB1 is present at high amounts in muscle and serum and undergoes oxidation in patients with MDs, contributing to the dystrophic phenotype by sustaining inflammation and muscle degeneration (6). IIMs correspond to a heterogeneous family of diseases with a chronic or subacute onset, involving immune cells and the injured tissue, recently divided into four more clearly defined clinical entities, namely dermatomyositis, inclusion body myositis, immune-mediated necrotizing myopathy, and antisynthetase syndrome (7). The HRS is a frequent target of autoantibodies in polymyositis/dermatomyositis: despite macrophages having a relevant and understood role in several genetically determined MDs, DCs seem to have a greater participation in IIMs. However, macrophages and DCs, together with T lymphocytes, are all innate-adaptive effectors through which muscle inflammation can worsen the pathology of both MDs and IIMs.

The majority of IIMs are firstly treated with immunotherapy but often secondary and also tertiary-line agents (chronic steroid-sparing immunosuppressive drugs, methotrexate, azathioprine, rituximab, immunoglobulins) are necessary to allow an amelioration of the pathological muscle signs (8, 9). Corticosteroids are recommended for treating Duchenne Muscular Dystrophy (DMD) where their use prolongs ambulation and life expectancy (10, 11). Despite this benefit, corticosteroid long-term use in DMD is associated with severe side effects, namely, adrenal suppression, growth impairment, poor bone health and metabolic syndrome. For other forms of MD like the limb girdle muscular dystrophies (LGMDs), corticosteroids are not typically used and only a few clinical trials and anecdotal evidences indicate that some forms of LGMDs may be responsive to steroids (10, 11). Intravenous immunoglobulins (IVIG) have been widely used in the treatment of autoimmune neuromuscular disease due to relatively few side effects (fever, myalgia, headache and nausea) and favorable therapeutic outcomes. Serious side effects, including thromboembolic events, renal failure, aseptic meningitis, and anaphylactic reactions, are rare and most concomitant to the increase in serum viscosity (12, 13). According to their anti-inflammatory properties and in particular to their ability to reduce significantly the activity of autoreactive T cells, IVIG were considered efficacious in combination with immunosuppressants and corticosteroids not only for primary immunodeficiencies but also for several inflammatory diseases (14).

Our goal in this review is to present current knowledge on the immunomodulatory function of immunoglobulins in genetic (MDs) and immune-mediated (IIMs) muscle diseases, exploring similarities and differences of the mechanisms by which immunoglobulins can modulate innate and adaptive immunity and to highlight areas in which further research is needed. There are already a number of excellent reviews on IVIG and neuromuscular diseases, but they are mostly focused on immune mediated muscle diseases and in particular Myasthenia gravis. The primary literature for review on each subject was derived from searching the National Center for Biotechnology Information PubMed database using the key words IGIV, IVIG, intravenous immunoglobulin, intravenous immune globulin, subcutaneous immunoglobulin, and subcutaneous immune globulin, along with key words specific for MD and IIM disease-related topic.

## Ig Link Innate To Adaptive Immunity

Immunoglobulins (Ig) are heterotetrameric glycoproteins formed by two identical heavy chains (HC) and two identical light chains (LC), linked together through di-sulfide bridge. Each Ig comprises a receptor-binding fragment (Fc, crystallizable fragment) that determines the isotype and two antigen-binding fragments (Fabs). Fc components of antibody are responsible for mediating the innate and immune response. FcγRs include activating receptors and one inhibitory receptor that are widely expressed in innate immune cells as neutrophils, natural killers, monocytes and macrophages while the component of adaptive immune system, as DCs and B cells, only express selected isoforms. The FcγRI isoform possesses high affinity for the IgG (15).

Five Ig isotypes exist: IgM, IgG, IgA, IgD and IgE that present different roles. The variability of antigen-binding sites is acquired thanks to genic rearrangement of HC gene segments: variable (V), diversity (D), and joining (J), while the V and J segments are recombined for LC chain. Variable domains are further variably joined thus this process produces a large antibody repertoire (16, 17). After antigen contact, somatic hypermutation increases variability and antigen affinity by introducing point mutations in the variable region. In a following step, cytokines stimulation and T cell interaction with Ig allowed the phenomenon of class switching, determining the change of Ig isotype, so that Ig acquired their specific function (18, 19). Fc structure will exert the formation of the immune complex between cells and their receptors: these interactions will determine signal-transduction effects, as opsonization of pathogens to favour their phagocytosis, activation of complement and antibody-dependent cytotoxicity (20).

## Ig Mediates Anti-Inflammatory Activity

The immunoglobulin found in highest concentration is IgG that is furtherly divided in four subclasses (IgG1, IgG2, IgG3, and IgG4), according to the length and number of di-sulfide bridges (varying their structural flexibility) and to different amino acid in Fc region (varying the affinity for Fc receptors). A core glycan is present in Fc structure allowing its modification by specific saccharide (21). Interestingly, glycosylation influences antibodies’ functions being the central mechanism of immune system activation. Of critical importance to such studies is the effect of glycosylated IgG on autoimmunity and immune-mediated tumor killing in cancer therapy (22). In this regard, it was demonstrated that glycosylation of IgG Fc fragment importantly modulated IgG phagocytic activity and cytotoxicity, thus affecting its anti-inflammatory activity. In addition, IgG hypoglycosylation was associated with inflammatory immune-response in rheumatoid arthritis and collagen-induced arthritis (23). Another form of post-translational non-enzymatic glycosylation is called glycation: it is accounted by glucose and free amino groups of lysine residues and determines the formation of advanced glycation end products (AGEs) (24). AGEs and their receptors (RAGE) are associated to different pathologies with important inflammatory background, such as atherosclerosis, diabetes and muscular disease (23). According to experimental evidences showing that the enrichment in terminal sialic acid of Ig residues could increase of more than 10-fold the anti-inflammatory activity of the preparation (25), modulation of glycosylation was used to develop therapeutic tools, as in autoimmune pathologies where high galactosylated/sialylated IgG antibodies blocked pro-inflammatory immune responses (26) or in rheumatoid arthritis, nephritis, lupus and sepsis (27).

Fab- and Fc-dependent mechanisms are presumed to be involved in the immunomodulatory effects of IgG (e.g., Ab neutralization, cytokines, complement molecules, blockade of neonatal Fc receptor and Fc activating receptors) (28). The Fab-dependent mechanisms diminished complement activation by neutralizing anaphylatoxins C3a and C5a and reducing the uptake of C3b and C4b onto the cell surface allowing neutrophils death (29). The Fc-dependent mechanisms block the formation of immune-complex and modulate DCs activation through FcγRIII thus inhibiting adaptive effector B cells (30). Moreover, primary Fc fragment of IgG sequence containing T cell epitopes for natural regulatory T cells (nTreg) is presumed to increase the nTreg population thus reducing the proliferative T cell response (31). The induction of these nTregs *via* IgG may have clinical implications to avoid immunogenicity and induce immune tolerance in MDs and IIMs.

## Immunoglobulin Medication

The therapeutic employ of immunoglobulin started over a century ago. Firstly, it was used to treat infections, later it became critical as a replacement therapy in immunoglobulin deficiency to provide passive immunity against pathogens (300 mg/kg, every 3 weeks). Nowadays IVIG gained critical importance as immunosuppressive strategies in autoimmune diseases, allowing administration of high doses (2 g/kg/month) in an easy way also in non-autonomous patients or in those with severe limitations in manual dexterity (32, 33). To date, FDA has approved IVIG for the treatment of different neurological conditions such as chronic inflammatory demyelinating polyneuropathy (CIDP) multifocal motor neuropathy (MMN), Guillan–Barré syndrome, myasthenia gravis, inflammatory myopathies but the request for off-label use of IVIG is increasing (13, 34–36). IVIGs are prepared by pooling more than 10,000 plasma or blood donations: thanks to donor heterogeneity, they consist of antibodies directed against a broad spectrum of pathogens and self-antigens (32). Recently, there was an increasing attention on subcutaneous injections of IgG (SCIG) as alternative route of administration. Different studies demonstrated at first in the primary immunodeficiency that SCIG resembled the same efficacy of IVIG, thus maintaining lower side effects; in addition, SCIG allows no need of costs of healthcare providers and hospitalization, improving patients’ quality of life (37). More importantly, patients that were refractory to IVIG well tolerated SCIG due to the slower absorption of IG (38). However, various drawbacks arose as the development of redness or swelling in the site of injection for continuous medications and the need of independence and self-reliance that is often lost or dramatically impaired in patients suffering for muscular weakness (37). To our knowledge, long-term studies on the undesirable effects of SCIG treatment have not been conducted.

## Similarities Between Muscular Dystrophy And Inflammatory Myopathy

The term myopathy can be applied to any muscle disease while the term dystrophy was classically applied by pathologists to the subset of genetic-dependent inherited myopathies in which muscle tissue destruction is the major feature. Despite etiological differences, genetic muscular dystrophy and immune-mediated inflammatory myopathy share many pathological features, namely, muscle degeneration, weakness, and different players in chronic inflammation as complement and other immune cells.

The study of innate and adaptive immune response involvement in MDs has been attracting the interest of many researchers though the results are so far barely exhaustive and sometimes contradictory. The temporal regulation of these processes is necessary to determine the differentiation of muscle progenitors into mature muscle fibers. In case of muscle injury and inflammatory events, DAMPs are released from necrotic muscle fibers and activate macrophages and DCs from the innate immune system, that in turn, elicits pro-inflammatory cytokines secretion and oxidative stress. These phenomena render the cells as APCs so that they can activate the CD4+/CD8+ T-lymphocytes from adaptive immune system, reinforcing the inflammatory cycle. Not surprisingly, all these cells from immune systems were isolated from the muscles of MD and IIM patients (as summarized in **Table 1**).

**Table 1:** Clinical and diagnostic peculiarities of muscular dystrophies and inflammatory myopathies.

Disease	Origin and pathogenesis	Cellular infiltrate	Muscle biopsies	Autoantibodies
***DMD***	Mutations in dystrophin gene	Neutrophils, M1 macrophaghes, mast cells (in early phase)	Muscle membranes ruptures	Anti-dystrophin
	Elevated CK	DCs, CD4+/CD8+ T-cells and Tregs (in late phase)	Complement activation	
	Cardiac and respiratory muscles dramatically affected		Necrosis of muscle fibers	
			Endomysial T cell infiltrate surrounding or invading necrotic muscle fibers	
			ROS and autophagy up-regulation	
			Fibrosis	
***LGMD-2R***	Mutations in dysferlin gene	Perimysial recruitment of macrophages and CD4+ T-cells	Marked lesions in muscle membranes	
	Pelvic and shoulder girdle muscle are preferentially affected		Myofiber degeneration and apoptosis	
			Abnormal activation of C4/C5 and down-regulation of CD55	
			Up-regulation of MHC-I	
***DM1/DM2***	Mutations in *DMPK* (DM1) and *CNBP* (DM2) Association with cardiac dysfunctions, insulin resistance cognitive impairment	Infiltration of CD4+ and CD8+ lymphocytes In both necrotic and non-necrotic muscles Upregulation of MHC-I expression	Fiber size variability, centrally-nucleated myofibers sarcoplasmic masses, atrophy and fibrosis	Anti-DM1
	Hypogammaglobulinemia			
***DM***	Proximal muscle preferientally affected	Macrophages, B-cells	Perimysial and vascular inflammation	Anti-MDA-5
		CD4+-T cells		Anti-Mi-2
	Skin rash	Plasmocytoid DCs	Perifascicular inflammation and atrophy	Anti- TIF1-γ
	Elevated CK			Anti-NXP-2
	Dysphagia		MHC-I and MxA expression on muscle fibers	
			MAC deposition on capillaries	
***PM***	Proximal muscle preferientally affected	CD28+ and CD8+ T-cells (in healthy muscle fibers)	MHC-I expression on healthy muscle fibers	Anti-synthetase
	Elevated CK	Monocyte-derived DCs	Perivascular inflammation	
		Macrophages		
***IBM***	Proximal and distal muscle affected	CD8+ T-cells (in healthy muscle fibers)	MHC-I expression on muscle fibers	Anti-cN-1A
	Muscle weakness in 50 years older patients	Macrophages in necrotic fibers	Autophagic vacuoles	
			Amyloid muscle deposits	
	Normal or slightly elevated CK	Myeloid DCs	Endomysial and perivascular inflammation	
		CD57+ NKs		
	Dysphagia			
***IMNM***	Proximal muscle preferientally affected Extremely elevated CK	CD68+ macrophages (in necrotic fibrs)	Necrotic muscle fibers MHC-I and MAC expression on healthy muscle fibers complement deposition on capillaries	Anti-SRP
	Severe weakness in adults	Paltry amount of CD4+/CD8+ T-cells and CD123+ plasmacytoid DCs		Anti-HMGCR

DMD, Duchenne Muscular Dystrophy; LGMD, Limb Girdle muscular dystrophy; DM1/DM2, Myotonic dystrophy 1 and 2; DM, Dermatomyositis; PM, Polymyositis; IBM, Inclusion body myositis; IMNM, Immune-mediated necrotizing myopathy; DCs, dendritic cells; NKs, natural killer cells.

However, clearly emerged that muscle biopsies of dystrophic patients showed more frequently diffuse variation of myofiber size, fiber hypertrophy, and myofiber fibrosis, while inflammation was considered as a response to cell damage. The inflammatory process leads to destruction of muscle tissue, that is often accompanied by muscle weakness, atrophy and necrosis, ending down in fatal fibrosis. On contrary, in inflammatory myopathies, the inflammatory development is not limited to muscles but it is frequently associated to vessels, and with a plethora of non-skeletal muscle clinical manifestations: fortunately, effective treatments for these diseases are available.

Similarities between MDs and IIMs might in some cases rise difficult the diagnosis; in fact, the presence of elevated creatine kinase (CK) levels, proximal weakness and muscle necrosis with perivascular infiltrates in muscle biopsy could be difficult to differentiate anti-SRP or anti-HMGCR myopathies from LGMD. Thus, fundamental insights for patients are provided by their clinical presentation together with the presence/absence of auto-antibodies and precise evaluation of the characteristics of muscle biopsies. Moreover, systemic disease manifestations (skin, lung), temporal pattern and steroid responsiveness are the parameters that are analyzed together to determine the diagnosis of patients thought to have a muscular dystrophy without family history or genetically confirmed diagnosis.

## Potential Ivig Targets For Mds Treatment

In MDs, the severity of muscle injury and inflammation dictates the impairment of muscle regeneration and successive replacement of myofibers with connective and adipose tissue (39) (**Table 1**). Among inflammatory mechanisms in dystrophic skeletal muscles, RAGE–NF-κB pathway and release of inflammatory cytokines could be potential targets for IVIG treatment. NF-κB system regulates gene expression in several cell types following inflammatory and immune responses: the up-regulation of NF-κB activity in human tissues during cumulative oxidative stress is strictly dependent on the amount of glycation end product, the N-carboxymethyllysine (CML), whose expression was showed in inflammatory diseases (40). Interestingly, CML are the well-known ligands of RAGEs that, in turns, stimulates autoimmune and/or chronic inflammatory cascades as MAPKs, Jak/STAT, PI3K (41). In general, the engagement of RAGE with its ligands (AGEs) elicit oxidative stress and trigger the inflammatory responses; however, the RAGE–NF-κB pathway is over-activated and involved in pro-inflammatory mechanisms in Duchenne Muscular dystrophy (DMD (42) and LGMD (43).

## Experimental Evidences Of Ig Benefits In Immunopathology Of Dmd

DMD is the most common, muscle-wasting disease of childhood and it is caused by mutations in dystrophin gene: the asynchronous cycles of muscle fiber degeneration exacerbate muscle infiltration of macrophages and lymphocytes and their secretion of pro-inflammatory cytokines. Although the primary defects rely on skeletal muscle structure, a multitude of secondary defects exist involving metabolic and inflammatory deregulated pathways. The skeletal muscle inflammation in DMD murine model (mdx mice) is a well-known and timely process that starts early in the first two weeks of life, has a peak at 6–8 weeks and decreases at 12 weeks. Neutrophils are the first cells to be identified in dystrophic muscles where they regulate the production of superoxide and myeloperoxidase and secrete TNF*α*: this condition allows membranes’ ruptures and, more importantly, fosters the recruitment of pro-inflammatory macrophages. The event that triggers the development of inflammatory phenotype is the invasion of M1 and M2 macrophages: the pro-inflammatory M1 secrete iNOS and cause the lysis of muscles while the pro-regenerative M2 regulate the activity of satellite cells. M1/M2 proliferation is regulated by the synergistic activity of cytokines (TNF-α, IFN-γ) and interleukins (IL4, IL10). Since in mdx mice the intracellular signaling pathways are chronically activated, the amount of pro-inflammatory M1 macrophages is dramatically deregulated with recruitment of adaptive effectors CD4/CD8+ T-cells (39, 44). Endomysial T cell infiltrate surrounding or invading necrotic muscle fibers were evident in DMD patients (45). In due course, it was demonstrated that dystrophin-specific T cells were present in some DMD patient’s blood (46) and further pathological analysis suggested that these cells could be primed by dystrophin positive revertants muscle fibers (47). In addition, the amount of dystrophin-specific T cell was directly dependent on the age of patients and, more interestingly, strongly reduced after steroid therapy (46). These studies in human subjects and other evidences coming from animal model of DMD (48–50) suggested that immunomodulation partly prevented the proliferation and priming of T-lymphocytes.

Similar study by Schmidt et al. (51) reported on the efficacy of IVIG (2 g/kg every 4 weeks for 2 months) in mdx mice to reduce the infiltration of cytotoxic T-lymphocytes and macrophages, leading to improvement of muscular architecture, endurance and muscular force. In addition, inflammatory muscle features were reduced as evidenced by down-regulation of CK levels and TGF-β, CCL2 and SPP1 (51). In line with these observations, Zschuntzsch et al. reported the successful long-term use of IVIG (2 g/kg every 4weeks for 18 months) of mdx mice: this therapy led to an improvement of cardiac and motor performance together with a significant reduction of macrophages and cytotoxic T-cells in addition to diminished amount of fibrosis (52). Together, these data underscore the role of the IVIG in modulating DMD pathological pathways at different levels through the inhibition of the activation and proliferation of T lymphocytes and reduction of the secretion of pro-inflammatory molecules.

**Figure 1:**
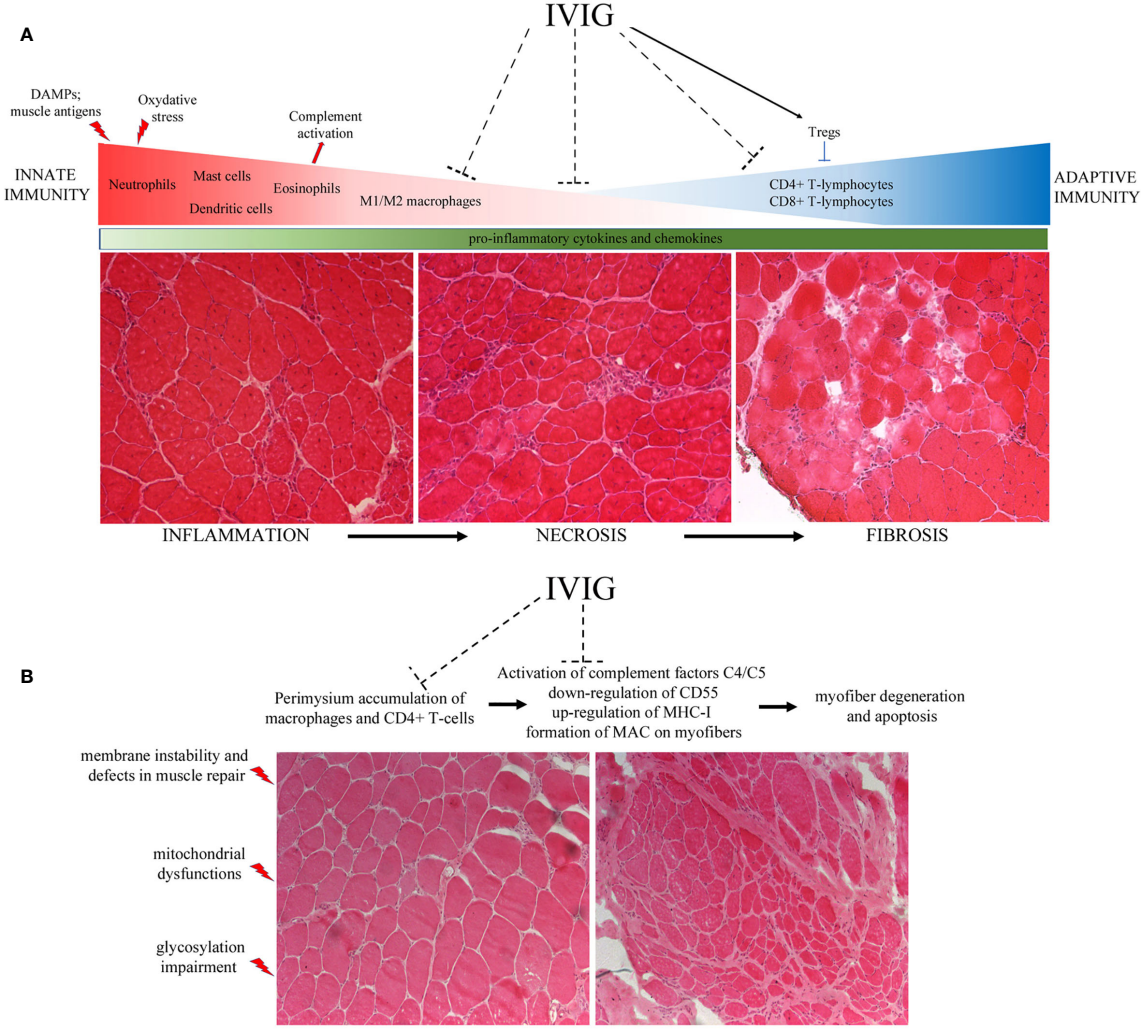
**(A)** The lack of dystrophin determines membranes’ instability and uncontrolled flux of cytoplasmic content into the extracellular matrix. These events lead to fiber destruction that contributes to chronic activation of the innate immune system. The release of DAMPs and the presence of muscle antigens activate first the neutrophils and immediately after the M1 macrophages and eosinophils. These cells secrete several pro-inflammatory cytokines and free radicals, causing the up-regulation of oxydative stress and the inflammatory *milieu* typical of DMD muscles. This condition provokes a second wave of inflammatory cell infiltration, mainly constituted by CD4+ and CD8+ cytotoxic T-lymphocytes. Unresolved inflammation causes the necrosis of fibers that ended down in the fibrosis of muscle tissues—often replaced by adipose cells—that are responsible for muscular weakness. **(B)** Mutations in the dysferlin gene are responsible for membrane instability and defects in muscle repair, causing mitochondrial dysfunctions. These phenomena determine the activation of complement system and the release of pro-inflammatory cytokines that in turn allow the recruitment of immune cells, prevalently macrophages around vessels and cytotoxic CD4+/CD8+-T lymphocytes in the perimysium. Taken together with the up-regulation of MAC and MHC-I on myofibers’ sarcolemma, the presence of such amount of inflammatory cells impairs muscle fiber necrosis.

Nunes et al. also reported the effect of IVIG treatment in ameliorating the muscle performance and survival rate of severely affected double knockout mdx/utr− dystrophic mouse whose myopathy is more similar to DMD patients (53). Interestingly, they evaluated the activation state of the dendritic cells (DCs) in the presence of co-stimulatory molecules as major histocompatibility complex (MHC), CD86 and CD40 and found that DCs activity was significantly inhibited in IVIG-treated mice, suggesting a role for IgG in the regulation of innate immunity. Accordingly, they described in treated animals lower amounts of pro-inflammatory IFN−γ, IL-1β and TNF−α, that were recently described as fundamental players for the activation of satellite cells (54) in muscle regeneration, wasting and development of fibrosis (53) (**Figure 1**).

In sum, animal models and mechanistic studies have shown promises in modifying the disease course of DMD: however, preclinical data are lacking in humans and informative trials with large patients’ cohorts are necessary to maximize beneficial outcomes.

## Ivig Clinical Evidences In Dysferlinopathy

LGMDs are a highly heterogeneous group of MDs with dominant and recessive forms, caused by different mutations that encompass over than 30 different genes. Among the LGDMs, the use of IVIG has only been explored in patients affected by dysferlin gene mutations (DYSF, MIM*603009) that are responsible for recessively inherited dysferlinopathy which is most pronounced in the pelvic and shoulder girdle muscles (Limb girdle muscular dystrophy R2, formerly LGMD2B), or distal myopathy with onset in gastrocnemius and soleus muscles in cases of Miyoshi myopathy (MM or MMD1), or distal myopathy with onset in the tibialis anterior (DMAT) (also referred to as DACM for distal anterior compartment myopathy) (55, 56). Dysferlin is a transmembrane protein implicated in protein vesicle fusion and trafficking and it is prevalently expressed in skeletal muscle but also in macrophages (57). Dysferlin also interacts with Ca^2+^ handling proteins for excitation-contraction (EC) coupling at the transverse-tubules (T-tubules) in skeletal muscle (58, 59). Thus dysferlinopathies suffer from devastating lesions in muscle membranes, leading to myofiber degeneration and enhanced recruitment of macrophages and CD4+ T-cells in the perimysium (60, 61),Confalonieri, 2003 #40462). Moreover, muscle fibers of both animal models and LGMDR2 patients present abnormal activation of complement factors C4/C5 together with the down-regulation of the CD55 complement inhibitory factor, the up-regulation of MHC-I and the formation of the membrane attack complex MAC (C5b-9) on their surface (62–64).

A clinical trial was launched to try to inhibit accumulation of complement deposit by the administration of IVIG in LGMD2R patients; however, the initially encouraging results were not pursued (65). Lerario et al. demonstrated increase of muscle strength for LGMD2R patients treated with Rituximab™ (a human/murine chimeric monoclonal antibody directed against CD20-positive B cells), suggesting a role of auto-antibodies in this pathology (66) (**Figure 1**). Nevertheless, these studies represent a starting point for IVIG testing trials in LGMD2R to alleviate the worsening of the disease in presence of complement deposit and auto-antibodies (67).

## Ivig Clinical Evidences In Patients Affected By Myotonic Dystrophy (Dm) With Hypogammaglobulinemia

The most common type of MDs beginning from adulthood is the Myotonic dystrophy, a multisystemic genetic disorder in which the inflammatory background determines the most evident muscular features, as fiber size variability, sarcoplasmic masses, atrophy and fibrosis (68). Other than muscular dysfunctions, DM develops several clinical presentations as cardiac dysfunctions (arrhythmias, atrial fibrillation, conduction defects), insulin resistance and cognitive impairment (69). Two forms of DM exist according to genes affected—type 1 (DM1) with mutations in the *DMPK* gene and type 2 (DM2) in the *CNBP* gene—and to phenotypic subtypes (70). Muscle biopsies from DM1 and DM2 patients shown high number of central nuclei and fiber diameter significant variation. According to the degree of muscular compromission, basophilic regenerating fibers, fibrosis and adipose development are evident in DMs patients. In particular, DM1 muscle biopsies are characterized by ring finger fibers and sarcoplasmic aggregates, while DM2 muscles show type-2 fiber atrophy—that are associated with severe muscular weakness—and possess a high number of centrally nucleated myofibers (71). Moreover, infiltrating CD4+/CD8+ T cells and over-expression of MHC-I were also observed (71). Hypogammaglobulinemia was found associated to DM1, especially for the IgG1 subclasses: it is believed that neonatal Fc receptor expressed on neutrophils and leukocytes (as in DCs) suffers from mutations causing IgG hypercatabolism. Alternatively, Suzumara et al. showed that the capillaries of DM1 patients were more permeable to IgG so that the enhanced extravascular IgG concentration was responsible for hypogammaglobulinemia (72). A reduced concentration of IgG1 was measured in several DM1 patients while IgA and IgM levels remained constant (73, 74). Intriguingly, increasing CTG repeat numbers were significantly correlated with decreasing serum IgG and IgG1 levels (75, 76) and lower concentrations of CD3+ and CD3+CD8+ lymphocytes (73). The study of Tieleman confirmed that DM2 patients’ sera had higher amounts of auto-antibodies related to DM1 and that this condition could lead to autoreactive T cell associated autoimmune diseases. Indeed, they suggested that DM2-causing mutations could dampen the immune functions of patients (77).

The evidence of IVIG usage in DM is limited to hypothesis stage as described in the study of Sasson et al. (78) where it was documented the clinical history of two DM1 patients (mother and son) with defects in circulating IgG related to kinetics and half-life: no defects in lymphocytes’ population were evidenced by cytofluorimetric FACS analysis nor other laboratory markers such as full blood count, electrolytes and urea were pathologic. Surprisingly, the patient with more significant serum IgG deficit had lower problems of infection. IVIG weekly treatment of these two DM1 patients leads to clinical stability for six months of follow-up (78). Although DM1-associated hypogammaglobulinemia represents a therapeutical indication, further studies are needed to determine the efficacy of IVIG to treat muscle symptoms in patients with DM1.

## The Immunobiology Of Idiopathic Inflammatory Myopathies

IIMs are the largest group of inflammatory muscle diseases that was recently classified in sub-categories: dermatomyositis (DM); polymyositis (PM); immune-mediated necrotizing myopathy (IMNM); inclusion body myositis (IBM) (79). Seronegative IMNM with increased risk of malignancy have been also described (79). The emerging characterization of myositis specific antibodies (MSA) associated to myo-pathological phenotypes has allowed to distinguish IIMs categories and new underlying pathomechanisms that should have therapeutic implications (8, 79, 80). Different evidences described in IIMs skeletal muscles—as T-cell mediated cellular infiltration, autoantibodies and MHC-I over-expression—prompted the researchers to define IIMs as immune-mediated muscle diseases. Accordingly, several immune components were identified as the main primary effector cells for each of IIMs—as the DC in DM, the CD8+-T cells in PM and IBM and the CD4+-T cells in IMNM. Thus, elegant studies on murine model of PM pointed out the fundamental role of DCs in combination with CD8+ cells in the pathogenesis of IIMs while other studies showed that genes involved in DCs activation, antigen-presentation and B-cell proliferation were up-regulated in IIMs canine models. Accordingly, the key pathological criteria for this classification are abnormal histological muscle features with presence of inflammatory cells and the suspects of autoimmune etiology, all being potential immunological targets of IVIG treatments (**Table 1**).

## Dermatomyositis

The clinicopathological classification for dermatomyositis was introduced by Dalakas in 1991. The recent discovery of prominent type 1 interferon (IFN1) signature and the recognition of dermatomyositis-specific antibodies (DMSA) determined current understanding of dermatomyositis classification (81). The clinical criteria for dermatomyositis included subacute or insidious onset symmetrical limb-girdle type muscle weakness and typical dermatomyositis skin rash (heliotrope rash, periorbital edema, Gottron papules, Gottron sign, V-sign, and shawl sign) (82). In dermatomyositis, the abundance of C5b-9 membranolytic-attack-complex on the endothelial cells together with C4d factor is responsible for capillary necrosis, leading to destruction of muscular fibers. Other features are perifascicular inflammation and atrophy of both type 1 and type 2 muscle fibers, reduced number of capillaries with MAC and immunoglobulins deposition on the endomysium (83). The activation of MAC is regulated by complement factor C1q that, in turn, allows the recruitment of inflammatory cells—mainly constituted by B-cells, CD4+ T-cells, plasmocytoid DCs and macrophages—while CD8+ T-lymphocytes and NK cells are extremely rare (84). Proximal muscles are preferentially affected and over-express IFN-1-dependent MHC-I and MxA proteins. DM are often associated with cardiac and pulmonary complications and with the presence of anti-MDA5, anti-NXP2, anti-Mi-2 and anti-TIF1-γ autoantibodies.

## Polymyositis

Polymyositis (PM) is an uncommon inflammatory muscle disease that preferentially affects women between 30 and 50 years old. The most common signs of PM are weakness of forearms, shoulders and back muscles with consequent difficulties in getting up, dry cough and dysphagia. Proximal muscles are invaded by CD28+ and CD8+-inflammatory T-cells that recognize muscular fibers expressing aberrantly the MHC-I (80). CD8+ T-cells include granules with perforin and granzymes, whose secretion upregulate the fibrosis of skeletal fibers. These phenomena determine the rising of endomysial/perivascular inflammation with monocyte-derived DCs together with absence of vacuoli, leading to muscle dysfunctions. In addition, TNF-α-related apoptosis-inducing ligand (TRAIL) protein, the inducer of autophagic cell death, is upregulated in both atrophic and regenerating muscular fibers and determines dysfunctions in NF-kB-mediated pathways and autophagy (85).

## Inclusion Body Myositis

The first classification of Inclusion body myositis (IBM) was done by Griggs et al. in 1995. The clinicopathological classification of IBM was later formulated into three categories: clinicopathologically defined IBM (CPD-IBM), clinically defined IBM, and probable IBM (86). The IBM is the most common form of IIMs in the adulthood: the inflammatory muscle features resemble those of PM and proximal and distal muscles are preferentially affected. The symptoms progress slower that the other IIMs and include weakness of finger muscles with consequently problems in gripping and buttoning and atrophy of forearms muscles. Pathological criteria require the presence of endomysial inflammatory cell infiltration, rimmed vacuoles, and presence of protein accumulation (by histochemical methods for amyloid or immunohistochemistry for SMI-31, or TDP-43). In addition to inflammatory myopathic changes, autophagic vacuoles expressing p62 and LC3 were also observed (8). Similar to PM, endomysial inflammation is driven by CD8+ T-cells and DCs, while macrophages are present in the necrotic fibers that are destined to phagocytosis. Antibodies anti-cytosolic 5’ nucleotidase 1A (cN-1A) are fundamental to discern IBM from the other IMNM and are responsible for the activation of B-cells. Recently, patients affected by IBM were associated to over-expression of CD8+ T-lymphocytes and CD57+ cells into myocytes (87). Unfortunately, immunotherapy and IVIGs are not effective for IBM patients with long disease (88, 89) whereas amelioration of muscle strength with decreased levels of creatine kinase were reported for short-term period (90). IVIG treatment is always recommended with more severe muscle weakness, bulbar, and respiratory involvement. Thorough more clinical trials and laboratory assessments including neurophysiology, antibody workup, muscle enzymes, and muscle biopsy are essential for the recognition and characterization of the IVIG mechanisms on IBM (91).

## Immune-Mediated Necrotizing Myopathy

IMNM is categorized into three subgroups according to positive antibodies: antisignal recognition particle (SRP) IMNM, anti3-hydroxy-3-methylgluaryl-coenzyme A reductase (HMGCR), and seronegative IMNM (92). IMNM can affect people of wide age range with acute onset of proximal weakness, whereas in children, the disease can be slowly progressed and mimic muscular dystrophy (93). Among the three subgroups, anti-SRP IMNM is associated with more severe muscle involvement and may associate with increased risk of cardiac involvement (94). Pathologically, IMNM is characterized by scattered necrotic and regenerating fibers with abundant infiltrating inflammatory macrophages and few perimysial/endomysial CD4+ and CD8+ T cells. On immunohistochemistry, C5b-9 is deposited on the sarcolemma of fibers showing mild HLA-ABC expression, suggesting that myofiber necrosis is mediated by antibody-mediated classical complement activation (95). Extra-muscular manifestations may also occur in IMNMs (79). IMNM patients are associated with anti-aminoacyl-tRNA synthetase (ARS) (anti-synthetase syndrome or ASS) and with anti-3-hydroxy-3-methylglutaryl-coA reductase (anti-HMGCR) and anti-signal recognition particle (anti-SRP) autoantibodies, that are not-pathogenic and whose function in antibody-dependent cell-mediated cytotoxicity (ADCC) process is still debating. However, Lega et al. described the clinical phenotype associated with ASS autoantibodies: it is characterized by perimysial and perivascular inflammation and important extent of necrosis; thus, it is often involved with other immune or connective diseases with anti Jo-1, PL-7, PL-12 antibodies (96). The anti-HMGCR and anti-SRP positive IMNM patients express MHC-I and MAC on non-necrotic sarcolemmal muscle fibers and present skin changes, arthralgia or synovitis, interstitial lung disease and myocarditis (79).

## Ivig Therapy For Iims

The first line of treatment for IIMs is the immunotherapy performed with prednisone and/or intravenous injection of glucocorticoids, and it is often associated to physiotherapy to recover muscle force. The IVIG is used as second line of treatment in steroids refractory dermatomyositis (32, 97), polymyositis and IMNM (98–100) patients, often with Rituximab in the case of IVIG are not efficacious. Of note, steroid free HMGCR-positive IMNM treated with IVIG showed reduction of CK levels and amelioration of muscle force without rescue of the pathophysiological myopathy (101). Cherin et al. demonstrated that SCIG treatment is well-tolerated and partly efficacious in steroid refractory IIMs patients (102). Similarly, other open-label studies in IIMs showed that SCIG partly ameliorated the myopathic symptoms without affecting the quality of life of patients (103, 104), diminishing the concentration of creatine kinases and resolving dysphagia (105, 106). Nevertheless, it became increasingly apparent the absence of randomized, blinded, controlled trails for IVIG or SCIG treatment in IIMs and the current knowledge in IIMs treatment is mostly empirical, based on experience of treating general features of IIMs.

## Recommendations And Concluding Remarks

The identification of dystrophin autoantibodies in DMD animal models (48, 107) and patients (46, 108) and the rising of IIMs-associated antibodies highlighted new pathomechanisms in the field of myology and suggested a possible role of the autoantibody-mediated activation of the complement cascade.

Most MDs and IIMs patients have a long disease duration with risk of relapse when corticosteroids or immunosuppressants are tapered or withdrawn. Taking into account the inhibitory and immune-modulatory features of IVIG, their involvement in mediating of different aspects of muscle pathologies gained importance in a clinical point-of-view. Immunoglobulins interact with complement cascade and ICAM-1 in muscle capillaries inhibiting the functions of macrophages and lymphocytes (8, 32, 79) leading to an overall reduction of pro-inflammatory cytokines/chemokines that sustain inflammation and consequent fibrosis, as in DMD (**Figure 1**). Although preclinical data from the literature indicate a pleiotropic effect of IVIG based on the reduction of innate and adaptive immune mechanisms, no clinical trials providing benefits of IVIG in MDs and IIMs patients have been reported so far and FDA-approval is still lacking. However, from a clinical perspective, the IVIG treatment of inflammatory myopathies do not produce reliable effects when administered alone as monotherapy and, more importantly, other severe pathological conditions as stroke and myocardial infarction get IVIG therapy extremely difficult. Recently, Lim et al. recently published a phase 2 open-label study in 20 IIM patients—with IBM not included in this cohort—to determine the efficacy and safety of three doses of IVIG (109). Unfortunately, only half of the patients exhibited moderate positive effects. Thus, direct evidences of the efficacy of IVIG approach in MDs and IIMs are still lacking.

Cost effectiveness of IVIGs in neuromuscular disease has also to be carefully evaluated. In fact, the cost of IVIGs is undeniably higher than corticosteroids and protocol for repeated assessments need to be developed to determine whether benefit is continued. Moreover, MD patients do not fit within strict homogeneous categories and individual responses can vary. In this sense, SCIG could represent an alternative administration of immunoglobulins allowing no need of costs of healthcare providers and hospitalization and improving patients’ quality of life. The future of IVIG therapy relies on better understanding of the mechanism of IVIGs treatment and on the possibility to engineer IVIGs preparation to allow targeted immunotherapeutic interventions in combination with mutation-based therapeutic efforts and to enhance their regulatory properties.

## Author Contributions

AF wrote the paper, prepared the original draft of the manuscript that was revised by YT. All the authors stated were involved in the critical revision of the manuscript and approved the final version of the article, including the authorship list. The corresponding authors had final responsibility for the decision to submit for publication. All authors contributed to the article and approved the submitted version.

## Funding

This study was supported by the Associazione Centro Dino Ferrari, a French Telethon AFM grant (No. 21104), by the Italian Ministry of Health (Ricerca corrente, FR230 distributed by Fondazione IRCCS Ca’ Granda Ospedale Maggiore Policlinico). This paper presents independent research funded by Ricerca Finalizzata 2016 (Linea di ricerca: “Theory-enhancing”).

## Conflict of Interest

The authors declare that the research was conducted in the absence of any commercial or financial relationships that could be construed as a potential conflict of interest.
